# Are There Lessons from India about the Management of Cattle? A Review of ‘*Cow Care in Hindu Animal Ethics*’ by Kenneth R. Valpey

**DOI:** 10.3390/ani11082175

**Published:** 2021-07-22

**Authors:** Clive J. C. Phillips

**Affiliations:** Curtin University Sustainability Policy (CUSP) Institute, Curtin University, Kent St., Bentley, WA 6102, Australia; clive.phillips@curtin.edu.au

**Keywords:** animal ethics, animal welfare, cattle, dairy cow, Hinduism, India

## Abstract

**Simple Summary:**

Cattle production has received significant criticism, particularly in Western countries, on account of its contribution to environmental pollution; the ethics of practices such as premature slaughter, particularly of male calves, and invasive procedures to control reproduction and dock tails and horns; and the poor welfare of cows in industrialised farming systems. However, in the country with the largest cattle population in the world, and one of the largest human populations, India, there is a culture of respect for cows, which has a long historical tradition. This culture is now growing to ensure that all cows are treated ethically. In his recent book, ‘*Cow Care in Hindu Animal Ethics*’, Kenneth Valpey describes the widespread advantages of such an approach, including a recognition that all beings are equal under a divine presence. The adoption of the Indian approach to cow care on a broader scale is considered, and it is recognised that whilst it generally provides for cow welfare and ethics better than Western farming systems, the adverse effects on the environment would be potentially magnified.

**Abstract:**

Cows are divine beings in Indian culture, a philosophy that is an important part of the Hindu faith. Although shared with other non-human animals, the focus on cows is well established in historical literature and is currently growing with a pattern of cow vigilantism in the country to try to enforce ethical standards in cattle keeping systems. The Indian attitudes to cows are considered in a new book by Kenneth Valpey, ‘*Cow Care in Hindu Animal Ethics*’. The content is highly relevant today, at a time when cattle farming is the subject of widespread concern in the West as a result of their contribution to environmental pollution; wasteful use of resources; and ethically questionable practices, such as male calf slaughter, reproduction control, and poor cow welfare. The contrast with systems predominating in the West, where cattle are essentially commodities from which products are obtained, is considered in this review of Valpey’s book. The development of a cow care culture, in which only cow milk surplus to her calf’s requirements is used for human consumption and cows are allowed to live out their natural life, is advocated. Whilst such a philosophy could usefully improve cattle care on a broad scale, the logical conclusion of extending human style citizenship to cows is that either human consumption of cattle products must reduce or more resources must be devoted to cows at the expense of other animals, including humans. There is evidence of the former, with a substitution of chicken meat for beef on a broad scale, and the prospect of laboratory-grown meat in the near future. In a small number of countries, meat consumption is already declining. These changes, coupled with a greater attention to cattle welfare practices, could herald a more ethical commensal relationship between cattle and humans in the future.

## 1. Introduction

Cattle farming is growing and intensifying in many parts of the world, in response to increasing demand for beef [1]. The model used for new dairy farms is usually one of intensive production on an industrial scale. Cows are permanently housed in groups of up to a few thousand and fed a loose mix of conserved forages and concentrated ingredients, despite advertising often suggesting to the consumer that the cows are kept on pasture. Calves are slaughtered at just a few days of age if they are male and reared to replace their short-lived mothers if female. Beef production in some of the world’s major beef producing countries, especially the USA and Australia, is equally intensive, with cattle finished in feedlots similar to the intensive dairy production model, or in the case of Australia, sent to Asia for finishing in feedlots. Other countries rear cattle primarily at pasture, for example Brazil, but the tendency is towards more intensive production systems, e.g., in China [2]. With this intensification has come criticism, mainly in Western countries, that cattle not only have poor welfare in the modern intensive production systems, leading to short lifespans, but they also contribute worldwide to climate change through their methane emissions, and they reduce food security through the increasing use of cereals in their diet, which could otherwise be used more efficiently to feed humans directly.

The world’s largest cattle herd is in India, where the predominantly vegetarian population rely on nutrients from dairy products as a major part of their diet. Cattle have been treated differently in India to the rest of the world for centuries, being revered and used mainly for dairy, not beef products, because of a belief that cow slaughter is immoral. A new book, ‘*Cow Care in Hindu Animal Ethics*’ by Kenneth R. Valpey, published in the Palgrave Macmillan animal ethics series, explores the evolution of the cow culture in India and what it means for society today, in India and elsewhere. In this review of Valpey’s book, I consider how Indian philosophy towards cattle management practices arose, and whether there are any lessons for cattle management in the rest of the world.

Valpey introduces the book by setting out the extent of the greater regard for cows in India than elsewhere. It is not just because of the majority Hindu population there, he argues, because the early writers on cow sanctity considered that they were making statements about cattle as a general principle, not just related to India or Hinduism. Nevertheless, attitudes towards cows are central to the Hindu faith. Given that there is general support for protection of cows in India, Valpey reminds us of the various motivations for this support. The least desirable is the uninformed, blind following of activist movements for cattle, often in association with membership of a particular political party, which may lead to hatred and violence. Such is seen in the cow vigilantism that has recently been spreading in India. Some vigilante groups patrol India’s borders to try to catch cattle smugglers, others lynch cow butchers or cow eaters (typically Muslims). Also undesirable is the call for action against cattle slaughterers that is borne from a desire to gain praise for oneself, a motive commonly associated with politicians. Most beneficial is the sort of dedicated activism, which seeks to make changes to the welfare and ethics of cattle production *for their sake*. In this regard, Valpey considers that cows should be considered as subjects in themselves, not objects with only instrumental significance, even though many people in India and perhaps most people in Western countries appear to adhere to the latter view. Here, there are lessons for today’s animal activists in Western countries.

## 2. Historical Literature on Indian Cow Culture

Valpey starts his discourse on South Asian literature on cow culture by acknowledging that domesticated cattle have been integral to the lives of people in this region for at least three millennia. Thus, we can expect, and indeed find, that cows are central to attitudes on fecundity, human possession of wealth, desirable living, indeed, in his words, the whole of cosmic order. Bulls have the added notions of virility and power.

The earliest of the sacred texts Valpey discusses, the *Rigveda*, was assembled well over 3000 years ago. It introduces the importance of cows, especially because of their provision of milk to be included in a drink, probably psychotropic, used in sacrificial rites. Indeed, cattle were at the centre of the life of the Vedic Aryans for whom the book was written. As a pastoral people, they needed to protect their cattle from predators and rustlers. To achieve this, they invoked sacrificial rites performed by brahmins, who were then the keepers of cattle. The hymns created for this purpose speak of a Vedic life revolving around three main elements: people, cows, and the natural world. Cows are seen as central to good human living; they provide succour, nurture, and the basis for a stable society. In so doing, they were a form of wealth in themselves. In the *Rigveda*, more apocryphal connections between humans and cattle are to be found, such as the similarity between cattle wandering off on their pastures and the human mind wandering during the sacrificial rites.

Moving on 500 years, a new genre of texts is introduced to take account of the transition to a more urban way of life, including the *Mahabharata*. This focusses on the pursuit of right behaviour, dharma, which includes the gifting of cows to bestow happiness on worthy individuals and, crucially, to obtain rewards in the next life. Another text considered is the celebrated *Bhagavata Purana*, which extols the life of Krishna, a cowherd who lived in a time of pastoral bliss, but who was also a supreme divinity in his own right, with great beauty and charm. His foibles were just minor mischief, such as stealing some yoghurt as a child and sharing it with monkeys, which is seen as a sign of his willingness to extend kindness to all beings. Valpey finally recalls several texts, mostly poems, that celebrate the passage of cows through the Middle Ages into their exalted position in modern times. These are mantras for remembering, praising, and protecting cows and their five main products: milk, yogurt, butter, dung, and urine. Even more recently, Indian gurus have placed meat eating alongside gambling, use of intoxicants, and illicit sex as the major crimes against society.

The conclusion of Valpey’s foray into Indian literature on sacred cows is that they are used to illustrate two sorts of polarity. The first is a contrast between cosmic order with clear and well-defined boundaries, dharma, and the desire to extend these boundaries, bhakti. Cows are symbols that mark the boundaries between the finite and infinite. The second polarity is between literal and figurative images, with cows spanning the divide between the physical, real world and the invisible world of divine beings and powers.

## 3. The Indian Cattle Care Model

Valpey proposes a model of animal ethics based on three Hindu paradigms—dharma, yoga, and bhakti. Although guidance to the right way to care for animals is provided by the Hindu religion, Valpey accepts, as Gandhi did, that it is not only relevant for Hindus, or even tied to specific castes in India. Dharma contributes a duty towards all living things, which is similar to the deontic and consequentialist normative ethics espoused by Western philosophers. Bhakti contributes a responsibility to individual beings, derived from a reverence for the divine Krishna, the simple cowherd, who tended animals, in particular his cattle, with great care and devotion. Yoga is the unifying force that recognises all beings as equal and facilitates self-cultivation to spiritual freedom.

Valpey advocates a simple cow culture for Hindu society, in which cows are hand-milked (to contribute positive human–animal relationships) by members of communal groups, with all members of the society supportive of this culture. Surplus milk only is offered to Krishna in daily devotional ceremonies and then to members of the community, most likely children, in meals taken communally. Such a culture would drastically reduce the amount of cows’ milk that is available for human consumption. Fewer calves would be available for beef production, and there is evidence of this, with a substitution of chicken meat for beef on a broad scale [3], and the prospect of laboratory-grown meat in the near future [4]. In a small number of countries, meat consumption is already declining [3]. The philosophy is in line with the sustainable village life espoused by Mahatma Gandhi. Valpey rejects abolitionist arguments that humans should not use animals in this way, believing that such a model constitutes a ‘world order of interdependence’. This model is highly preferable to the parasitic way of life based on extracting as much as possible from animals, in a model based solely on economics, that has become the Western norm. Furthermore, he believes that such positive interaction between humans and animals contributes to personal development and a closer relationship to the deity. In so doing, they contribute to four virtue-nourishing practices—compassion, austerity, purity, and truthfulness. This communitarian model recognises cattle as citizens, which the state should advocate in support of communal good over and above individual’s rights.

Will this model work in an India that is currently refocusing itself on an industrialised future, leaving Gandhi’s land of model villages, with their inhabitants spinning their way to eternity? India’s dairy farmers have been modernising by crossing their cows with high-yielding Western breeds [5], especially the Friesian, which necessitates feeding a high-energy diet, which in turn requires intensive fertilising of their land to produce more crops.

Would it work outside of India to counteract the criticisms of global cattle production that it utilises food and water resources wastefully, that it is responsible for a large part of greenhouse gas emissions, that it pollutes watercourses and the sea, that the animals are subject to widespread cruelty in their short lives? To date, about 84 cow farms around the world have been developed on the communitarian model, most with just 5–10 cows. The productivity of these cows is a fraction of that given by cows in the West, so if adopted on a global scale, either the world would need more cows or intake would have to be, as Valpey proposes, restricted to those most in need, i.e., infants. Certainly, the world could do without the enormous quantities of dried cows’ milk fed to infants, since both extended breast feeding and vegetable-based milk replacer, based usually on soya or pea protein, are viable alternatives for most infants. Vegetable-based milk replacers are not always quite as efficiently digested [6], but are already widely used for raising calves, with their mother’s milk being used to raise human infants instead. What about the social function that Valpey perceives for cattle, as humans tend cattle in a close relationship? Admittedly, the demeanour of cattle lends itself to provision of such social support: they are docile, long-suffering, and readily strike up a relationship with their human carers. Indeed, it is their very vulnerability that made them suitable as a symbol for change from the aggressive oppression challenged by the likes of Gandhi. In addition, they have the added advantage of being herbivorous, avoiding the necessity to be fed other animals, unlike, for example, cats, which as obligate carnivores are generally fed the by-products of our animal industries. Cats are therefore intrinsically linked to industries that are increasingly viewed with suspicion from an ethical standpoint. However, in their role as companion animals, cows have a number of disadvantages, not least their size, which brings large feed requirements and risks of harm, particularly to children. Although generally docile, both males and occasionally females can be aggressive, particularly if confronted with our other major animal companion, dogs. The desirability for these animal relationships will be questioned in future. Do we have absolute requirements for interactions with domesticated animals? Clearly many humans do not and go through their lives oblivious to the animal kingdom around them. More empathetic humans generally extend their nurturing to animals as well as humans, and dogs and cats currently appear to fulfil that important role in their lives. We have a different but related need to connect to wild animals, whether this entails supporting their preservation in the wild or direct interaction in zoos or, preferably, their natural habitats. The Indian example of cows communing with their human keepers should make us question our future relationships with domesticated animals.

What of the accusation of widespread cruelty in modern, industrialised cow production? Would this be any less in Valpey’s model? The cruelty largely stems from the excessive demands for high levels of milk production and the failure of the cows’ metabolism to cope, leading to high rates of disease, excessive weight loss, and failure to reproduce. Cows typically last only two years in the most intensive systems [7]. In the model systems that Valpey describes, which have been tested on a small scale in several Western countries, cows live well into their teens. However, in such systems there are concerns about artificial control of reproduction and castration of males, both of which are generally accepted without question in industrialised dairy systems. Similarly in Indian gaushalas, where cows are retired after being released from their lives as dairy producers, cows live until their mid-teens and are generally well fed [8]. The main welfare concerns in the gaushalas are overcrowding, lack of access to pasture, and poor-quality flooring. Gaushalas reduce the opportunity that cows have for natural behaviour, with no access to pasture for grazing, separation of the sexes, and no offspring for cows to nurture. India as a nation has less than 4% of her land as pasture, and increasingly cattle are fed only by-products, with no grazing and only a little grass harvested daily, being fed to the animals in barns [9]. The large numbers of cows turned loose by dairy farmers after they are no longer productive overwhelms the gaushala system, and increasingly the facilities to house the growing number of dairy cows retired there are inadequate. The matriarchal group that forms the basis for cattle living in the wild is not possible in gaushalas but might be possible on a small scale in Valpey’s communitarian model, which he calls an ‘ahimsa dairy’.

## 4. Welfare vs. Environmental Impact?

This model of cattle management has most to commend it in terms of animal welfare and ethics, and least in terms of managing the impact of cattle on the environment, both local and global. The ahimsa dairies can be seen as offering improved care to cattle. Calves are given free access to their dam’s milk; oxen are not overworked in the fields; and, in the interests of maintaining fairness, those suspected of not managing cows appropriately are dealt with through the courts of law and not by activists themselves. The accounts of cow-caring enterprises are openly available. The liberty of people to eat meat and other animal products should be respected by cow care activists, Valpey suggests, although activism to reduce meat consumption is encouraged. If meat is to be consumed, Valpey controversially suggests that it should be meat that has been produced through some sort of religious sacrifice, in this case according to Hindu texts, by the appropriately qualified person and sanctioned by the state. Parallels with halal and kosher slaughter are obvious.

The importance of cow carers being knowledgeable in their work has been recognised since the writing of the *Bhagavad Gita*, since this protects the cows from betrayal by their carers and, ultimately, commodification, according to Valpey. Increasing people’s knowledge of ethically sensitive issues improves their empathy and ability to recognise animal emotions [10]. The importance of training is recognised, as it was by Gandhi, to farmers in particular, but also in schools and colleges [11]. The training should acknowledge the need to deeply reform human–environment interactions and be based on organic farming practices. It will help to create a culture of respect for cows that will discourage illegal activities that deny this respect, such as smuggling cattle to other countries for slaughter, which is rife in India at the present time. Adherents to cow care philosophy should have a culture of respect for all living beings and a humble recognition of all life being in connection with a divine being, symbolised by Krishna, the playful cow carer.

Valpey concludes with a pessimistic forecast that for all the philosophising about cow treatment, humans as cow predators will remain as such, unless their fundamental attitude to cow care changes. It is futile to suggest that consumers in the West will not pay for better cow care when one of the world’s poorest countries has this tradition of care that transcends anything offered elsewhere. The fact that cow care in India does differ from that in the West demonstrates that some good comes out of the religious association of cows with divine beings. However, although India embraces, in Valpey’s words, ‘care-full attention’ to cow welfare and ethics, the sustainability of ahimsa dairies from an environmental perspective is questionable.

## Data Availability

There is no data for this review.
